# Assessing the Prevalence of Incidental Findings Identified by CTPA in Women of Reproductive Age

**DOI:** 10.1155/2018/4630945

**Published:** 2018-11-04

**Authors:** Nigel Champion, Sarah Hogan, Jeffery Flemming

**Affiliations:** Department of Radiology and Nuclear Medicine, Memorial University of Newfoundland, St. John's, NL, Canada

## Abstract

**Background and Objective:**

Though multiple studies have evaluated the prevalence of incidental findings identified by CTPA, none have done so with a focus on reproductive-age females with normal chest X-ray (CXR). Due to a comparatively lower breast radiation dose, the oft-recommended alternative to CTPA in this patient group is a V/Q scan. However, these are limited in their assessment of these alternate findings; therefore, it is of particular importance to evaluate the likelihood of these findings on CT in this patient group, which is the goal of this study.

**Methods:**

Through a review of our PACS system, female patients aged 18-50 years who underwent diagnostic CTPA prior to April 1, 2017, were identified. The 100 most recent cases which had a normal CXR within 48 hours of CTPA were included. Incidental/non-PE findings were then divided into PE-positive (PE+) and PE-negative (PE-), and subcategorized into types I, II, III, and nil non-PE finding groups. Type I findings required immediate follow-up or intervention, type II findings required outpatient follow-up, and type III findings required no follow-up or were previously known.

**Results:**

PE was detected in 15% of scans. Type I findings were found in 8% of patients (0% of PE+, 9.4% of PE-), type II findings in 10% of patients (13.3% of PE+, 9.4% of PE-), type III findings in 34% of patients (40% of PE+, 32.9% of PE-), and nil non-PE finding in 48% of patients (46.7% PE+, 48.2% of PE-).

**Conclusion:**

While CTPA identifies incidental findings in the majority of patients, a small minority of these findings are likely to alter immediate management. In the context in increased radiation risk, this strengthens the argument that alternate imaging modalities such as V/Q should be strongly considered for the investigation of potential PE in women of reproductive age with normal CXR.

## 1. Introduction

Computed tomographic pulmonary angiography (CTPA) is the most commonly used imaging modality for the identification of pulmonary embolism (PE) [1, 2]. As access and accuracy have increased, there has been a steady rise in the popularity amongst ordering physicians [1–3], as well as the number of incidental findings made by CTPA [1, 3]. In other words, findings other than PE discovered by CTPA which may or may not explain patient symptomatology are a common occurrence [1, 3]. While the identification of both PE and non-PE findings in one may be beneficial to patient care, this must be weighed against the potentially harmful effects of overtesting. Though CTPA may offer an alternate explanation for patient symptoms, it also comes with the risk of potentially unnecessary follow-up procedures and contrast nephrotoxicity, as well as the risk of increased ionizing radiation exposure as compared to alternative imaging modalities [4–6].

One population that has been identified as particularly at-risk for excess radiation exposure is women of reproductive age [4, 6]. There is evidence demonstrating a heightened radiosensitivity in this denser breast tissue, conferring added risk for excessive CT use in women of reproductive age [4, 6]. For this reason, ventilation-perfusion (V/Q) scanning has remained an oft-favored imaging modality in this patient population [4, 7]. The radiation dose delivered to breast tissue in CTPA has been shown to be many times higher than V/Q on average and though the radiation dose delivered to the uterus is slightly higher in V/Q than in CTPA, both have been shown to be very small and likely less significant in comparison to the breast dose [4].

A conundrum often encountered by clinicians is whether the chances of identifying non-PE pathology justify the use of CTPA over V/Q. Several studies have assessed the prevalence of incidental/non-PE findings made by CTPA in the general population [8–13], with the discovery of relevant non-PE CT findings ranging from 7.6% to 57% of scans, depending on categorization schemata [8–13]. None of these studies have however focused on women of reproductive age who otherwise have a normal chest X-ray, which is an important segment of the general population, as there is an alternative method for the diagnosis of PE that is often suggested for these patients, namely, a V/Q scan, which has the primary shortcoming of not being able to identify these alternative findings. This study was performed to aid in the clinical decision-making process for these patients.

## 2. Methods

### 2.1. Setting and Population

All female patients aged 18 to 50 years who underwent CTPA at one center prior to April 1, 2017, were considered for inclusion. Through a review of the PACS system, the examinations of the abovementioned patients were identified and reviewed for inclusion and exclusion criteria, regardless of indication for CTPA, laboratory markers of thrombosis, or pretest probability scores. Only final reports which had been signed-off by a Royal-College-certified radiologist were included. Only patients with normal chest X-ray within 48 hrs prior to undergoing CTPA were considered, as to minimize the inclusion of findings which did not necessitate CT for discovery, and with the aim of ensuring findings identified in this study were truly the benefit of CT alone. The most recent 100 patients meeting criteria were included, thus making the date range of included studies to be June 22, 2015, to March 28, 2017.

Data was collected from the Health Sciences Center in St. John's, NL, Canada. Images were acquired using a Toshiba Aquillion One CT scanner via helical acquisition with slice thickness of 2 mm, pitch 0.813, 100 kvp, and AIDR. The contrast media was 60 mL of iopamidol 370mgl/mL at 5cc/s.

### 2.2. Data Collection

The study was approved by our local health research ethics board (HREB). Select data were obtained from PACS system and recorded without identifiers by two medical students. Data collected from records included CXR findings and date, CTPA findings and date, and whether the study was positive or negative for PE. Data relevant to the calculation of radiation dose was also collected from department records.

### 2.3. Data Analysis

The categorization of patients was based on identified or suggested pathology, as well as suggestions for follow-up written in the reporting radiologists' final reports. To mitigate potential heterogeneity as a result of professional opinion, abnormal scans were reviewed by the research team's Royal-College-certified radiologist. In cases of disagreement between the reporting radiologist and the diagnostic or follow-up opinion of our reviewing radiologist, a third Royal-College-certified radiologist was asked to offer determination. This was done with a preference given for the most potentially harmful diagnosis interpreted by the primary or study radiologist(s). The classification scheme used was that proposed by Perelas et al. [13], as adapted from Richman et al. [11]. This scheme was found during a review of available literature and chosen as it appeared to be the most comprehensive and methodically similar study to that which we aimed to perform. The categories are as follows:

- **Type I findings**: requiring immediate intervention or inpatient monitoring. This included but was not limited to findings suggestive of infection, new malignancy, severe inflammation, or pneumothorax.
- **Type II findings**: not requiring immediate intervention, but necessitating outpatient follow-up. This included but was not limited to nonspecific ground-glass opacities, pulmonary nodules (based upon Fleischner Society guidelines [14]), lymphadenopathy, and pleural effusions.
- **Type III findings**: not requiring any intervention or follow-up. This included but was not limited to minor atelectasis, previously known pathology, stable pulmonary nodules (based upon Fleischner Society guidelines [14]), and benign osseous lesions.
- **Nil non-PE findings**: no pathology identified by CT.

In the case of multiple findings, patients were categorized based upon the most significant finding with the most significant follow-up requirement.

## 3. Results

The most recent 100 patient records which met the criteria of having a normal chest radiograph as well as a diagnostic CTPA were identified. Of these patients, PE was detected by CTPA in 15 (diagnostic yield of 15.0%). Patients were stratified into PE-positive and PE-negative groups, with subcategorization into type 1, type 2, type 3, or nil non-PE finding groups (Table 1)

Amongst the 15 patients with positive studies for PE, no type I findings were identified. Type II findings were present in 2 PE-positive patients (13.3%), including one ground-glass nodule requiring follow-up and one small pleural effusion (Table 2). Type III findings were present in 6 PE-positive patients (40.0%), including 3 cases of atelectasis, 1 case of osteoarthritis, 1 case of small parenchymal hyperattenuation, and 1 case of pulmonary infarct/hemorrhage secondary to PE (Table 2). No incidental non-PE finding was discovered in 7 PE-positive patients (46.7%) (Table 1).

Amongst the 85 patients with negative studies for PE, 8 patients had type I findings (9.4%), including 6 cases of pneumonia, 1 case of bronchiectasis combined with pneumonia, and 1 case of pneumomediastinum (Table 2). Type II findings were present in 8 PE-negative patients (9.4%), including 5 cases of ground-glass/pulmonary nodules and 1 case of lymphadenopathy which necessitated follow-up imaging as per Fleischner Society guidelines [14], as well as 2 small pleural effusions (Table 2). Type III findings were present in 28 PE-negative patients (32.9%), 12 of whom had atelectasis on CT (Table 2). The remainder of type III PE-negative findings included calcified granulomas, lymphadenopathy, pulmonary nodules not requiring follow-up based on Fleischner Society guidelines [14], bony abnormalities requiring no intervention or follow-up, a pulmonary cyst, a goiter, and a previously known infection (Table 2). No incidental findings were detected in the remaining 41 PE-negative patients (48.2%) (Table 1).

In total, type I findings were found in 8 (8%) of scans, type II in 10 (10%), type III in 34 (34%), and nil non-PE finding in 48 (48%) (Table 2).

## 4. Discussion

Comparison of PE-positive and PE-negative groups was limited due to the total number of charts reviewed. It is apparent, however, that the distribution of each finding type within either group is similar, with nil-findings being the most common, followed by types III, II, and then I findings (Table 1). While there were no type I findings amongst PE-positive patients, this may simply be that it is unlikely to develop two symptomatic pathologies concurrently in an otherwise relatively healthy population. Though the comparison is again limited by the number of reviewed charts, type II findings are largely comprised of ground-glass/pulmonary nodules in either group (Table 2). Unsurprisingly, the most common non-PE finding identified in either PE-positive or PE-negative groups by CTPA was isolated atelectasis (Table 2).

Upon analysis of the PE-negative group and with the exception of one case, all type I findings were suggestive of potential early infection. However, the findings of infection in these cases were ultimately nonspecific. While radiologic evidence may have been helpful in the diagnoses of some cases, a presumptive diagnosis and empiric therapy may have been possible for many, assuming symptoms were present in addition to other clinical and laboratory findings. Conversely, these findings are undoubtedly not clinically relevant for many patients, potentially leading to unnecessary treatment.

The majority of type II findings in the PE-negative group were nonspecific ground-glass opacities and pulmonary nodules. Based on Fleischer Society guidelines [14], these would require eventual follow-up to rule out malignancy after discovery. The use of CT for cancer screening is a contentious topic however, considering the potential additional cost and morbidity of following up these nodules which in the vast majority of cases will turn out to be benign, and the identification of chronic nodules is certainly not an indication for CT in an acute setting.

Thus, the majority of patients without PE (51.7%) had non-PE finding(s) on CTPA that were not apparent on CXR. Despite this, combining type III and nil-non-PE finding groups would indicate that 81.1% of scans did not alter management in any way, and the remaining 18.9% required some form of intervention or follow-up. If lung nodules are excluded, this leaves a maximum of 12.9% of non-PE findings in this study sample which could potentially explain the symptomatology of a PE and alter immediate management.

Comparison with similar, previous studies is difficult, as there was a wide variability of study designs amongst available research in this area. While some studies have yielded rates as low as 7.6%, others have demonstrated rates as high as 57% or above [8–13]. This is due, in part, to what individual researchers considered to be significant findings and also whether CTPA was compared to other modalities such as CXR.

When considering only studies with comparable finding categorization schemes, the results of this study are similar, however. Richman et al., who had a comparable study design to that which we used, found that 7% of their study population required immediate intervention and 10% required eventual follow-up for a combined 17% as compared to our 18% [11]. Chandra et al. reviewed 12640 CTPAs at one center, determining that 7.6% of all CTPAs revealed previously unknown findings of clinical significance (previously unknown from clinical or other investigative techniques) [12]. This was further reduced in this study to 3.2% when patients with low to intermediate pretest scores were excluded however, which we were unable to account for due to study design [12]. Based on criteria used in this study, the former value may be more comparable to our 12.9% of non-PE CTPA findings, though lower.

Perelas et al. found that 80.1% of their 580 PE-negative patients had findings on CTPA, which was much more than our 52% [13]. However, only 14.3% of scans in total could explain symptomatology, and these values did not account for CXR [13]. When analysis was limited to the 578 patients who had both CXR and CTPA performed, only 3.1% had intrathoracic pathology which was visualized on CT but not CXR [13]. This was comparatively lower than our 12.9%.

Overall, this study is distinct from most previous studies in that (1) our study population is limited to women of reproductive age rather than all-comers and (2) only patients with normal chest X-ray prior to CTPA were included in our study, which limits the inclusion of non-PE findings which may have been identified by X-ray alone as well as selects for a population which is eligible for alternate imaging modalities such as V/Q. These differences may account for the different values we found as compared to our predecessors.

This study is, however, limited by the number of reviewed charts, with inadequate power to compare PE-positive and PE-negative groups. Generalizability is difficult as this was a single-center study. It is also possible that pathology visible on X-ray could have developed within the up-to-48-hour window between CXR and CTPA in some patients, meaning some CTPA findings in this study could have been falsely represented. As only abnormal scans were reviewed by reviewing radiologists, some findings could theoretically have been missed in reported “normal” scans. Importantly, long-term outcomes were not tracked, implying an inability to interpret findings in the context of clinical diagnosis, or eventual morbidity or mortality.

Among reviewed studies, PE detection rates ranged from 9% to 24.2% [8–11, 13], as compared to our 15%.

## 5. Conclusion

While CTPA identifies non-PE pathology in the majority of patients without PE, most of these findings do not alter immediate patient management. Given both the heightened risk of radiation exposure in this population and the baseline risks associated CT and subsequent follow-up testing, this strengthens the argument that alternate imaging modalities such as V/Q should be strongly considered for the investigation of potential PE in women of reproductive age with normal chest X-ray.

## Figures and Tables

**Table 1:** Findings of CTPA categorized by PE detection and finding types, represented as proportions of PE-positive or PE-negative findings, respectively. Finding types are also represented as a proportion of all findings, independent of PE detection on the right side.

**Finding category**	**PE-negative**		**PE-positive**		**Total**
	**n**	%** of PE (+)**	**n**	%** of PE (-)**	%** of total by type**
Type I	8	9.4%	0	0.0%	8%
Type II	8	9.4%	2	13.3%	10%
Type III	28	32.9%	6	40.0%	34%
Nil non-PE	41	48.2%	7	46.7%	48%

**Table 2:** Incidental findings made by CTPA, categorized by PE detection status (positive or negative) and finding type (types I, II, and III), represented as number (n) of said finding. When multiple pathologies were identified, incidental findings were categorized based on the most immediately harmful pathology.

**PE-negative**			**PE-positive**		
**Finding category**	**Finding**	**n**	**Finding category**	**Finding**	**n**
Type I	Pneumonia	6	Type I		
	Bronchiectasis & Pneumonia	1			
	Pneumomediastinum	1			
					

Type II	Ground-glass/pulmonary nodule(s)	5	Type II	Ground-glass/pulmonary nodule(s)	1
	Lymphadenopathy	1		Pleural effusion	1
	Pleural Effusion	2			
					

Type III	Atelectasis	12	Type III	Atelectasis	3
	Calcified granuloma(s)	3		Pulmonary infarct/hemorrhage	1
	Lymphadenopathy	3		Small parenchymal hyperattenuation	1
	Pulmonary nodules	2		Osteoarthritis	1
	Osteoarthritis	2			
	Vertebral compression fracture	1			
	Healed fracture	1			
	Hepatic steatosis	1			
	Pulmonary cyst	1			
	Enlarged thyroid	1			
	Previously known infection	1			

## Data Availability

Data to support the findings of this study, beyond that which is included in this manuscript, are available from the corresponding author upon reasonable request.
